# Bilateral Vocal Cord Paralysis Following Influenza A: Case Report

**DOI:** 10.1002/ccr3.71737

**Published:** 2026-01-12

**Authors:** Dan H. V. Tran, Xiang Lay, Winston Cheung, Blake Lindsay, Benjamin Worrall, Sarika Suresh, Arun Aggarwal, Mark Kol, Atul Wagh, Rosalba Cross, Asim Shah

**Affiliations:** ^1^ Intensive Care Department Concord Repatriation General Hospital Concord New South Wales Australia; ^2^ Anaesthetic Department Concord Repatriation General Hospital Concord New South Wales Australia; ^3^ Ear Nose and Throat Department Concord Repatriation General Hospital Concord New South Wales Australia; ^4^ Neurology Department Concord Repatriation General Hospital Concord New South Wales Australia

**Keywords:** bilateral, cord, influenza A, palsy, paralysis, vocal

## Abstract

Bilateral vocal cord paralysis (BVCP) is a rare but potentially life‐threatening condition. Viral infections such as COVID‐19, herpes simplex virus, Epstein–Barr virus, and cytomegalovirus have been widely reported; however, influenza A is a rarely reported cause. We describe a 57‐year‐old male who developed BVCP following an influenza A infection. Comprehensive investigations, including neuroimaging and nerve conduction studies, ruled out structural, neoplastic, demyelinating, medication‐related, and autoimmune causes. A transient CMV viremia was identified but deemed a secondary phenomenon due to immunosuppression. The patient required intubation, intravenous corticosteroid therapy, and ICU care. Vocal cord function gradually improved over several weeks, with complete resolution at 2 months. The temporal relationship, exclusion of other etiologies, and spontaneous recovery are very consistent with a post‐viral inflammatory mechanism secondary to influenza A. Clinicians should maintain clinical suspicion for bilateral vocal cord paralysis following influenza A for future practice.

## Key Clinical Message

Our consensus identified influenza A as the most likely cause of this patient's BVCP despite confounding factors such as multiple myeloma, CMV viremia, and pomalidomide exposure. Clinicians should remain vigilant, as influenza‐related BVCP may occur even with comorbidities and provided other potential etiologies are appropriately excluded.

## Graphical abstract

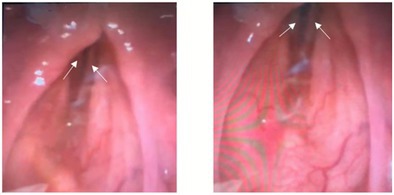

## 1 Introduction

Bilateral vocal cord paralysis (BVCP) is a rare, life‐threatening condition that can compromise airway patency [1]. COVID‐19, herpes simplex virus, Epstein–Barr virus (EBV), and cytomegalovirus (CMV) have been widely reported as potential viral causes [1, 2, 3]. Influenza A has been associated with neurological syndromes such as brainstem encephalitis and Guillain‐Barre syndrome; however, historically there have only been two reports of vocal cord paralysis secondary to influenzal origin [4, 5].

We present a case report of a 57‐year‐old male who developed BVCP following influenza A infection.

## 2 Case History

A 57‐year‐old male presented with dyspnoea, cough, fevers, and coryzal symptoms. He was receiving isatuximab, pomalidomide, and dexamethasone in four‐weekly cycles for refractory lambda light chain multiple myeloma. His blood profile was neutropenic on presentation, and he was placed on piperacillin‐tazobactam. This was switched to oseltamivir for 5 days when influenza A was isolated on a respiratory PCR swab. His admission was complicated by a bacterial pneumonia that was empirically managed with ceftriaxone and doxycycline. Supplemental oxygen was administered via low‐flow nasal prongs and the fraction of inspired oxygen (FiO_2_) did not exceed 28%. His fever, dyspnoea, and cough improved but he had an ongoing wheeze that was being treated with prednisolone.

On day 21 of admission, he developed an audible inspiratory stridor that was associated with new voice hoarseness, dysphonia, accessory respiratory muscle use, paradoxical breathing, and difficulty speaking in full sentences. There was no facial or neck swelling, and his lung fields were clear to auscultation. There was no focal neurological deficit on examination. The patient was placed on high‐flow nasal prong (HFNP) oxygen at an FiO_2_ of 100% and flow rate of 50 L/min.

## 3 Investigations

**FIGURE 1:**
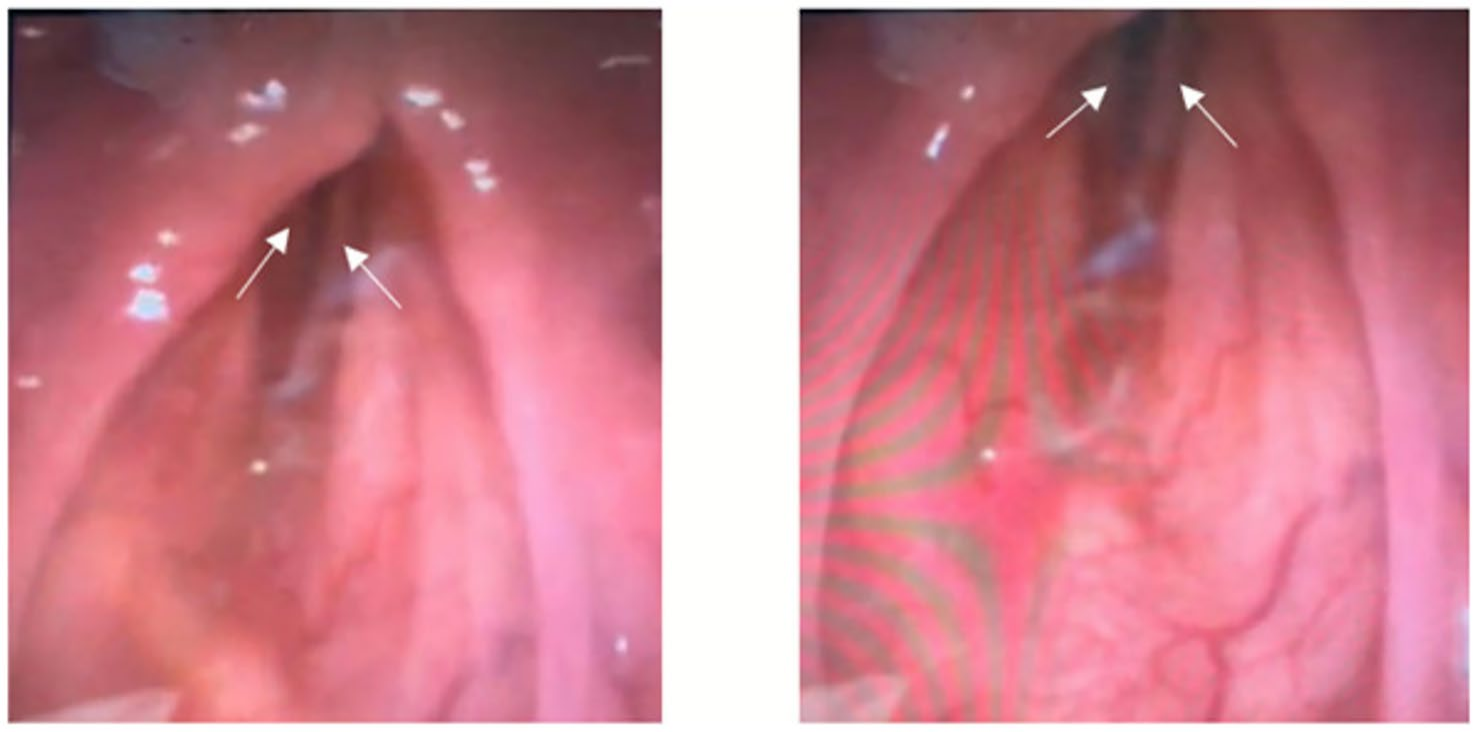
Flexible nasal endoscopy demonstrating bilateral vocal cord paralysis (BVCP) in the paramedian position. The left image demonstrates a glottic gap between the vocal cords (white arrows) in the maximally adducted position (patient saying “eeee”). Under normal circumstances the vocal cords should be touching with no glottic gap. The right image shows his vocal cords (white arrows) during inspiration (the maximally abducted position), which has only minimally moved in comparison to the left image. The vocal cords should be 9 mm away from the midline on full abduction [6].

Flexible nasal endoscopy (FNE) demonstrated BVCP in the paramedian position with no vocal cord or supraglottic edema (Figure 1).

He was intubated and a 5‐day course of intravenous dexamethasone commenced. On day 1 following intubation, he was kept on light sedation with propofol and fentanyl. Examination of cranial nerves II–VII and XI was unremarkable, with assessment of other cranial nerves limited due to the endotracheal tube. He had normal tone in the upper and lower limbs, no clonus, and normal power and reflexes in all muscle groups.

Computed tomography (CT) scan of the brain demonstrated no intracranial hemorrhage, mass, or other abnormalities other than stable lytic lesions in the mandible, base of skull, and cranium. A CT scan of the neck demonstrated cervical spine stenosis at C5‐6, with multiple myeloma‐related degenerative changes, but no pathology affecting the recurrent laryngeal nerves.

**FIGURE 2:**
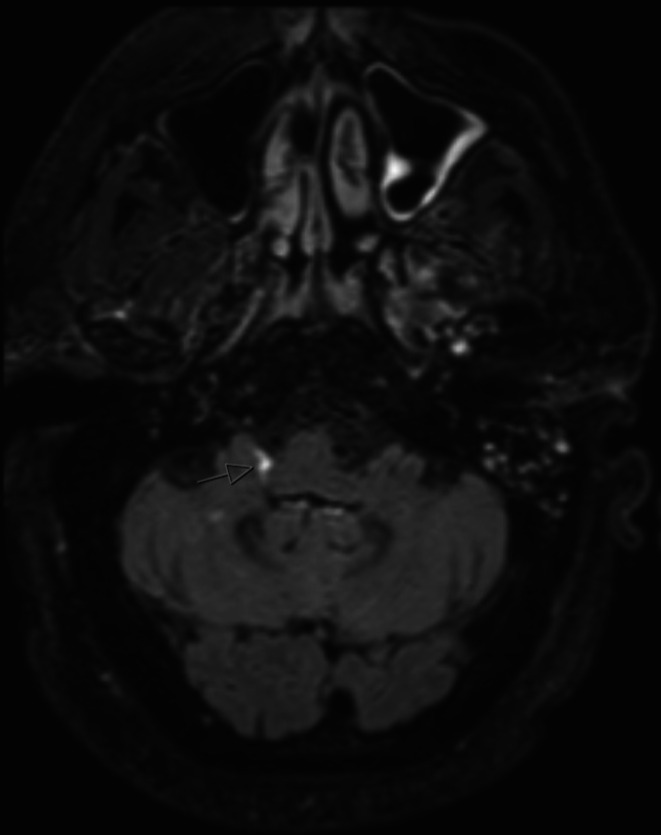
MRI brainstem post gadolinium T2 FLAIR demonstrating leptomeningeal enhancement (arrow) on the right side of the medulla extending into the root of the vagus nerve.

Magnetic resonance imaging (MRI) of the brain and brainstem (Figure 2) demonstrated the presence of leptomeningeal infiltration around the medulla at the site of the vagus nerve predominantly on the right.

The cerebral spinal fluid (CSF) did not demonstrate any major abnormalities other than albuminocytologic dissociation (Table 1).

**TABLE 1:** Lumbar puncture results.

Test	Result	Reference range
General lumbar puncture results		
CSF protein	0.79 g/L (high)	0.20–0.62 g/L
CSF glucose	4.3 mmol/L	2.5–4.5 mmol/L
CSF lactate	2.1 mmol/L	1.1–2.8 mmol/L
CSF appearance	Clear and colorless	
CSF CMV	Not detected	
CSF culture	Negative	
Autoimmune encephalitis screen		
CSF NMDA Receptor AB	Not detected	
CSF CASPR2 Ab	Not detected	
CSF LGI‐1 Ab	Not detected	
CSF GABA‐B Ab	Not detected	
CSF DPPX Ab	Not detected	
CSF IgLON5 Ab	Not detected	

The patient's full blood count, electrolytes, and liver function tests were within normal limits. Immunological blood tests including a multiple myeloma screen, as seen in Table 2, were mainly unremarkable apart from his CMV DNA titers, which started to rise to 23,000 copies per mL after ICU admission. This was believed to be a viremia secondary to his ICU admission (with previous known infection) and corticosteroid immunosuppression rather than a cause for his vocal cord paralysis.

**TABLE 2:** Immunology results.

Test	Result	Reference range
Antineuronal antibodies		
Anti‐MAG antibodies	Negative	
Anti‐GM1 (IgG and IgM) antibodies	Negative	
Anti‐GQ1b (IgG)	Negative	
Multiple myeloma screen		
Free kappa light chains	13.7 mg/L	6.7–22.4 mg/L
Free lambda light chains	53.6 mg/L (stable for 2 months)	8.3–27.03 mg/L
Kappa/Lamba ratio	0.26 (Low)	0.31–1.56
Beta 2 microglobulin	2.1 mg/L	0.8–2.4 mg/L
Other immunology		
Alpha 1	2.0 g/L	1.0–2.8 g/L
Alpha 2	10.0 g/L	5.4–10.0 g/L
Beta globulin	9.0 g/L	6.1–12.0 g/L
Gamma globulin	8.0 g/L	6.4–15.0 g/L
Immunoglobulin A level	0.89 g/L	0.7–3.12 g/L
Immunoglobulin G level	10.8 g/L	6.39–15.6 g/L
Immunoglobulin M level	0.4 g/L	0.5–3.0 g/L
Antinuclear antibodies	Not detected	
ENA	Not detected	
Neutrophil cytoplasmic antibodies	Not detected	
ANCA	Not detected	
GM1 ganglioside IgM antibodies	Not detected	
Muscle specific kinase antibodies	Not detected	
CMV quantitative DNA	Over 2 weeks, peaked at > 23,000 copies per mL then down trended to 2000 per mL on discharge	
Serum cryptococcal antigens	Not detected	
Rheumatoid factor	< 16 IU/mL	

The patient was extubated after 3 days. FNE showed mild vocal cord edema likely secondary to prolonged intubation. Vocal cord movement had returned but was not back to baseline. His stridor, dysphonia, and work of breathing had significantly improved.

Dexamethasone was restarted after finishing the 5‐day course as the patient redeveloped worsening stridor, voice hoarseness, and increased work of breathing. Examination of his neurological reflexes, cranial nerves, and peripheral motor and sensory function were normal. A nerve conduction study was unremarkable.

**FIGURE 3:**
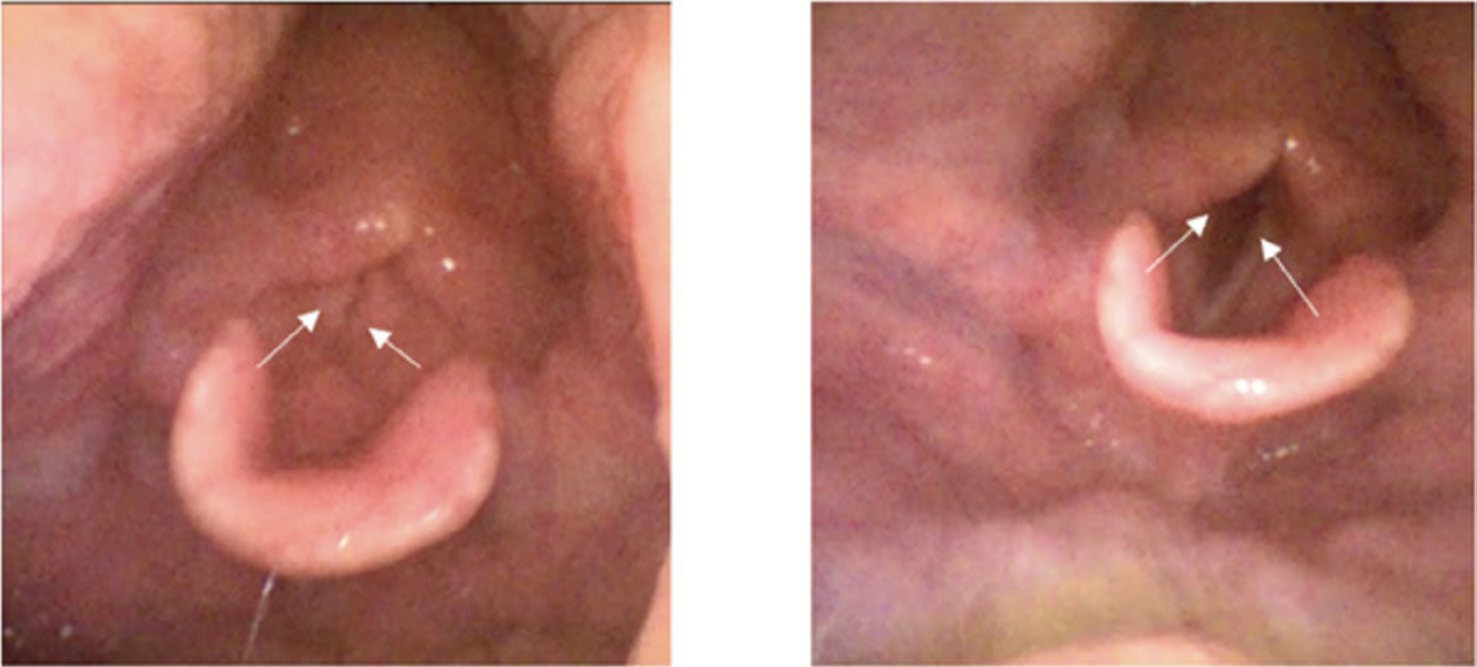
Flexible nasal endoscopy demonstrating improved vocal cord movement. The left image shows the vocal cords (white arrows) in the adducted position (patient saying “eeee”) and the right image shows the vocal cords (white arrows) in the maximally abducted position (patient during inspiration). In comparison to Figure 2, there is now complete closure of the vocal cords on adduction and significantly more vocal cord abduction (although still not in the full abducted position 9 mm away from midline). There are normal aryepiglottic folds/pyriform fossae with minimal supraglottic edema.

FNE on day 29 demonstrated improved vocal cord adduction and minimal supraglottic oedema, albeit with still limited vocal cord abduction (Figure 3).

After ICU stepdown, IV valganciclovir was started for 1 week for the CMV viremia, then stepped down to oral valganciclovir on discharge. At discharge, his CMV quantitative DNA result was 2250 copies/mL.

Repeat FNE on day 37 demonstrated improving bilateral mobile vocal cords with only ongoing limited abduction.

Repeat MRI of the brain and brainstem demonstrated stable leptomeningeal enhancement greater around the right foramen of Luschka. This was now recharacterized as an asymmetrical choroid plexus rather than acute pathology. There was no vagus or glossopharyngeal nerve involvement. The patient was discharged on day 41 with soft biphasic stridor on deep inspiration and no cranial or peripheral neurological deficits.

## 4 Outcome and Follow Up

His vocal cord function returned to normal after 2 months.

## 5 Discussion

In this case, influenza A was identified as the most likely cause of the patient's BVCP, following an extensive workup to exclude more common etiologies. Although the exact pathophysiological mechanism underlying viral‐mediated vocal cord paralysis remains unclear, it is hypothesized to involve neuronal inflammation triggered by an immune response to viral infection [4].

FNE did not reveal any structural or anatomical abnormalities, and neuroimaging excluded central neurological causes including meningeal carcinomatosis. Comprehensive serological and CSF analyses ruled out infectious, demyelinating, and autoimmune processes. Furthermore, nerve conduction studies provided additional evidence against a demyelinating neuropathy. The patient had no history of laryngeal trauma, head or neck malignancy, or surgical intervention, and there was no use of alcohol or recreational drugs to suggest toxic neuritis as a potential cause.

The recharacterization of the MRI findings as “asymmetrical choroid plexus” were agreed upon by multiple neuroradiologists and the conclusion was made in the clinical knowledge of the patient's improving symptoms amidst the stable unchanging imaging findings around the vagus nerve. Regardless, the unilateral enhancement would not be able to explain the observed bilateral pathology. The absence of MRI abnormalities does not specifically rule out a viral or immune‐mediated inflammatory neuropathy, as these processes can occur with normal neuroimaging early in their course or when affecting structures below the resolution of routine MRI [7]. The unremarkable MRI helped to exclude other central causes of bilateral vocal cord palsy, such as neoplasm, mass effect, or brainstem pathology.

Consideration was also given to a paraneoplastic neurological process related to multiple myeloma. Central nervous system involvement, including toxic‐metabolic encephalopathy or central nervous system myelomatosis, was initially contemplated; however, such entities typically manifest with subacute cognitive decline, multifocal neurological deficits, limb weakness, and abnormalities in speech or gait, rather than isolated cranial nerve involvement [8]. Paraneoplastic neuropathies in plasma‐cell dyscrasias are rare and more commonly present as progressive, length‐dependent sensorimotor neuropathies or demyelinating syndromes, rather than an isolated bilateral recurrent laryngeal nerve palsy [9, 10].

Furthermore, a recent case presentation in the *American Journal of Respiratory and Critical Care Medicine* described vocal cord paralysis in a patient with multiple myeloma, in whom the neurological course was characterized by a preceding and worsening mononeuritis multiplex, established peripheral neuropathy, and lack of response to corticosteroids [11]. This phenotype aligns with recognized paraneoplastic patterns in plasma‐cell malignancies [8, 9, 11]. In contrast, our patient demonstrated no sensory disturbance, limb weakness, autonomic dysfunction, or painful neuropathy. Peripheral nerve conduction studies were normal, and a comprehensive antibody panel was negative (although it is acknowledged that some myeloma‐associated paraneoplastic antibodies were not specifically assayed) [12]. Additionally, serial neuroimaging revealed no definitive evidence of leptomeningeal or neural infiltration, and myeloma markers remained biochemically stable throughout the illness.

Taken together, the absence of peripheral neurological features, normal electrophysiological and immunological studies, stable myeloma burden, and the lack of definitive radiological CNS involvement strongly argue against a multiple‐myeloma–associated paraneoplastic syndrome as the cause of this patient's isolated bilateral vocal cord palsy.

Reactivation of CMV in this patient is unlikely to have been the cause of his BVCP. Instead, it more plausibly represents an opportunistic viremia in the context of immunosuppression from dexamethasone, superimposed on a background of CMV seropositivity. Extensive literature describes a high incidence of CMV reactivation in critically ill ICU patients, largely attributed to severe illness and immunosuppressive states [13, 14].

**FIGURE 4:**
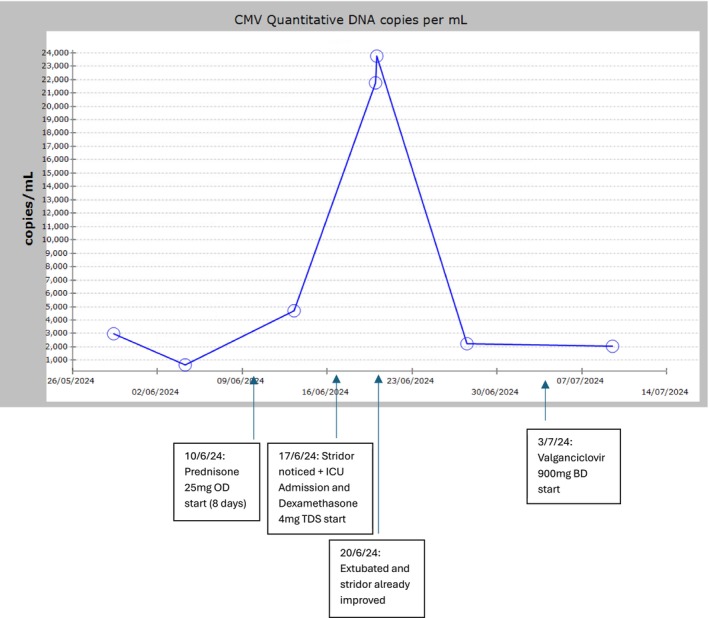
Graph demonstrating the temporal relationship of steroid initiation, CMV titer peaking, ICU admission, symptom course, and valganciclovir initiation during CMV reactivation.

Several key factors support the interpretation that CMV reactivation was a secondary phenomenon rather than the primary driver of this patient's bilateral vocal cord palsy. Notably, CMV DNA levels began to rise only after the initiation of corticosteroid therapy and ICU admission, consistent with stress and immunosuppression‐associated reactivation. Peak viremia occurred on Day 3 of ICU admission; however, by this stage the patient had already demonstrated clear clinical improvement after extubation, indicating that neurological recovery was underway prior to this despite increasing viral titers. Although a negative CSF CMV PCR does not entirely exclude CMV neuroinvasion, there were no clinical features of CMV end‐organ disease, such as enterocolitis, hepatitis, or polyradiculopathy, which would typically be anticipated at this degree of viremia in true CMV neurological involvement [15]. Importantly, valganciclovir therapy was commenced only later in the admission, well after the patient's neurological improvement had occurred, further supporting that CMV reactivation was incidental and temporally unrelated to his clinical course. Figure 4 highlights these events on a timeline.

Moreover, previously reported cases of CMV associated BVCP have described patients with a more prolonged and systemic course, including symptoms such as pharyngitis, fever, and marked poly motor and sensory neuropathies developing over several months before the onset of BVCP [3, 16, 17, 18].

Pomalidomide, an immunomodulatory agent, has been associated with neurotoxicity and has been cited in rare instances as a potential cause of central cranial neuropathies. A review of previously reported cases of central neurotoxicity linked to immunomodulatory drugs identified symptoms such as reversible coma, amnesia, dysarthria, and lethargy [19, 20]. In contrast, our patient exhibited none of these features, and serial MRI imaging demonstrated no central craniopathy corresponding to the bilateral vocal cord palsy. Moreover, there were no additional central neurological deficits, and pomalidomide therapy continued throughout the period of clinical improvement. Collectively, these factors make pomalidomide‐related central neurotoxicity an extremely unlikely cause in this case.

Although peripheral neuropathy has been reported as a potential adverse effect of pomalidomide, we were unable to identify any definitive published case reports establishing a direct causal association. In this patient, peripheral nerve conduction studies were normal, further arguing against a pomalidomide‐related peripheral neuropathy as the underlying mechanism.

The cerebrospinal fluid demonstrated albuminocytologic dissociation, characterized by elevated protein (0.79 g/L) in the presence of a normal white cell count. This pattern is classically associated with immune‐mediated inflammatory neuropathies, such as Guillain–Barré syndrome or acute disseminated encephalomyelitis (ADEM), where disruption of the blood–nerve barrier permits protein leakage without corresponding pleocytosis [21, 22]. In this case, the absence of encephalopathy, peripheral neuropathic symptoms, and normal peripheral nerve conduction studies make Guillain–Barré syndrome, ADEM, and related peripheral demyelinating processes unlikely. Furthermore, this CSF profile helps to exclude bacterial, viral, or carcinomatous meningitides, which would typically demonstrate pleocytosis or other inflammatory markers [23]. In the context of this patient's recent viral illness and clinical presentation, albuminocytologic dissociation therefore provides supportive evidence for a post‐viral, immune‐mediated inflammatory mechanism, rather than a direct infectious or structural neuropathic process.

The recurrence of symptoms following dexamethasone cessation further supports that the pathology likely relates to some inflammatory cause. The dexamethasone could have played a role in reducing either post‐extubation edema or viral‐mediated neuronal inflammation.

The acute, post‐infective onset of symptoms, absence of definitive cranial or peripheral neuropathy, and responsiveness to steroid treatment are consistent with the limited existing reports of BVCP secondary to influenza infection [1, 4, 5].

Overall, our multidisciplinary consensus identified Influenza A as the most likely cause of the patient's BVCP, despite the various confounding factors including multiple myeloma, CMV viremia, and pomalidomide exposure. We recommend, where relevant, clinicians maintain clinical suspicion for this for future practice, as influenza A‐related BVCP can occur even in the setting of confounding factors if other etiologies for the BVCP have been appropriately explored.

## Author Contributions

**Dan H. V. Tran:** conceptualization, writing – original draft, writing – review and editing. **Xiang Lay:** writing – original draft, writing – review and editing. **Winston Cheung:** supervision, writing – original draft, writing – review and editing. **Blake Lindsay:** writing – original draft, writing – review and editing. **Benjamin Worrall:** writing – original draft, writing – review and editing. **Sarika Suresh:** writing – original draft, writing – review and editing. **Arun Aggarwal:** supervision, writing – original draft, writing – review and editing. **Mark Kol:** supervision, writing – original draft, writing – review and editing. **Atul Wagh:** supervision, writing – original draft, writing – review and editing. **Rosalba Cross:** supervision, writing – original draft, writing – review and editing. **Asim Shah:** supervision, writing – original draft, writing – review and editing.

## Funding

The authors have nothing to report.

## Ethics Statement

This case report has received ethical approval by the Sydney Local Health District Research Ethics and Governance Office.

## Consent

Written informed consent was obtained from the patient for the publication of this case report.

## Conflicts of Interest

The authors declare no conflicts of interest.

## Data Availability

All pertinent data are included in the article. Further inquiries can be directed to the corresponding author.
